# Freeze-Drying Effects on Viability and Cellular Stability in a Subset of Sourdough Lactic Acid Bacteria Strains

**DOI:** 10.1007/s00284-025-04673-5

**Published:** 2025-12-11

**Authors:** Valentina Musi, Elisa Aiello, Mattia Pia Arena, Luciana De Vero, Andrea Pulvirenti, Maria Gullo

**Affiliations:** Department of Life Sciences, University of Modena and Reggio Emilia, Reggio Emilia, 42122 Italy

## Abstract

Microbial resources are essential for food biotechnology, where they contribute to product quality, process efficiency, and safety. Their long-term preservation is critical for culture collections (CCs) and microbial biological resource centers (mBRCs). This study investigates the effects of freeze-drying on five lactic acid bacteria (LAB) strains from the Unimore Microbial Culture Collection (UMCC), comparing non-lyophilized and lyophilized cultures under two pre-freezing protocols: overnight incubation at -20 °C (PF-20) and 2-hour incubation at -80 °C (PF-80). Viability, fermentative performance, metabolite production, and cell integrity were assessed using microbial counts, HPLC profiling, and SEM imaging, respectively. *Lactiplantibacillus plantarum* UMCC 2996 maintained over 95% viability and stable fermentative traits across all conditions. In contrast, *Fructilactobacillus sanfranciscensis* UMCC 2990 and *Leuconostoc citreum* UMCC 3011 showed significant viability losses (down to 54.45% and 73.89%, respectively) and altered metabolic profiles, particularly under PF-80. SEM analysis confirmed structural damage in sensitive strains, with visible cellular debris and membrane wrinkling. This study provides novel perspectives on the customized assessment of freeze-drying protocols for sourdough-derived LAB strains with industrial potential, confirming the need for tailored preservation strategies to ensure the long-term functionality of LAB strains in CCs and mBRCs.

**Supplementary Information:**

The online version contains supplementary material available at 10.1007/s00284-025-04673-5.

## Introduction

Microorganisms are the backbone of fermented foods, directly influencing factors such as fermentation time, product nutrition, taste, and biosafety [1–5]. Among them, lactic acid bacteria (LAB) are essential to various food processes, including the fermentation of products such as yogurt, cheese, and sourdough, where they enhance flavor, texture, and shelf life [6]. LAB are Gram-positive bacteria that primarily utilize carbohydrates as their main carbon source [7]. They are typically cocci or rods and exhibit strong tolerance to low pH environments. Recently, the family *Lactobacillaceae* has undergone significant taxonomic revision, resulting in the reclassification of the genus *Lactobacillus* into 25 distinct genera [8, 9]. By producing lactic acid, LAB acts as natural preservatives -suppressing spoilage and pathogenic microbes - while also improving sensory qualities and probiotic functionality in fermented foods [10–18]. Sourdough exemplifies a complex fermentation ecosystem dominated by LAB and yeasts. Although present in lower numbers, yeasts thrive in the acidic environment created by LAB and contribute to dough leavening [19]. Within sourdough, strains belonging to the genera *Lactiplantibacillus (L.)*, *Fructilactobacillus (F.)*, *Furfurilactobacillus (Fu)*, *Pediococcus (P.)*, and *Leuconostoc (Le.)* are commonly found. *Fructilactobacillus. sanfranciscensis* is often the predominant LAB species, and the resulting diverse microbiota influences human health benefits [20] and drives technological performance, affecting bread’s nutritional value, sensory profile, shelf life, and overall quality [19, 21, 22]. Preserving LAB viability, stability, and functional traits is vital for their industrial application. Culture collections (CCs) and microbial biological resource centers (mBRCs) play a central role by storing strains and curating associated genomic, transcriptomic, proteomic, and metabolomic data to ensure reproducibility and accessibility [23]. These biological repositories support research, education, screening assays, biotechnological development, and patent protection [24, 25]. To maintain genetic integrity and long-term stability, CCs and mBRCs employ multiple ex-situ preservation strategies, such as sub-culturing, storage under mineral oil or in sterile soil, immobilization on carriers, silica gel, or spray-drying, desiccation, vitrification, cryopreservation, and freeze-drying [26]. International guidelines from the World Federation for Culture Collections (WFCC) and the OECD recommend applying at least two complementary methods per strain [27]. Among these, cryopreservation and freeze-drying are the most reliable for broad-spectrum, long-term maintenance [24]. Freeze-drying offers advantages such as room-temperature storage, reduced transportation costs, and high recovery of viable cells when optimized with appropriate cryoprotectants and lyophilization conditions. However, strain-specific responses to preservation require optimization of parameters such as suspension media, lyoprotectant type and concentration, cell density, pre-freezing temperature, and cooling rate. These factors influence survival rates by mitigating ice crystal formation, osmotic stress, and protein denaturation [28–30]. The use of lyoprotectants further enhances survival rates by stabilizing cell structures [31–33]. Rigorous validation of these parameters is crucial to ensure reproducibility, purity, identity, and retention of industrially relevant traits, including fermentative capacity, antimicrobial activity, and exopolysaccharide production, over extended storage periods [34].

The aim of this study was to assess the effectiveness of freeze-drying, as second preservation method of five LAB strains belonging to five different species and all isolated from sourdough (Table 1). To this purpose cell viability, morphology and fermentative functionality, were evaluated before and after freeze-drying, using two different protocols.

**Table 1:** Lactic acid bacteria strains used in this study (De Vero et al. 2021; Iosca et al. 2022; Iosca et al. 2023a; Iosca et al.^ 2023^b)

UMCC code	Species	Isolation source^a^	Industrial traits	NCBI 16 S rRNA Accession Number
UMCC 2990	*Fructilactobacillus sanfranciscensis*	Sourdough type I	Demonstrated anti-mould activity, including complete inhibition of *Fusarium graminearum*; significant inhibition of *Aspergillus flavus*; antibacterial activity against *Escherichia coli*, *Campylobacter jejuni*, *Salmonella typhimurium*, and *Listeria monocytogenes*; exopolysaccharide production.	MZ170706.1
UMCC 2996	*Lactiplantibacillus plantarum*	Dough for Panettone	Demonstrated anti-mould activity, including complete inhibition of *Fusarium graminearum* and *Aspergillus niger*; significant inhibition of *Aspergillus flavus*; broad antibacterial activity against *Escherichia coli*, *Campylobacter jejuni*, *Salmonella typhimurium*, and *Listeria monocytogenes*; exopolysaccharide production; putative anti-spoilage LAB with high inhibitory activity.	MZ170701.1
UMCC 3002	*Furfurilactobacillus rossiae*	Dough for Panettone	Demonstrated anti-mould activity, including complete inhibition of *Fusarium graminearum* and *Aspergillus niger*; significant inhibition of *Aspergillus flavus*; exopolysaccharide production; good bio-preservation activity against *Aspergillus flavus* ITEM 7828 in bakery products; promising candidate for valorization of food waste and by-products in a circular economy perspective.	MZ170709.1
UMCC 3010	*Pediococcus pentosaceus*	Gluten-free sourdough	Demonstrated anti-mould activity, including complete inhibition of *Fusarium graminearum* and *Aspergillus niger*; moderate inhibition of *Aspergillus flavus*; antibacterial activity against *Escherichia coli*, *Campylobacter jejuni*, *Salmonella typhimurium*, and *Listeria monocytogenes*; exopolysaccharide production; putative anti-spoilage LAB with high inhibitory activity.	PX068364
UMCC 3011	*Leuconostoc citreum*	Dough for Panettone	Demonstrated anti-mould activity, including complete inhibition of *Fusarium graminearum* and *Aspergillus niger*; moderate inhibition of *Aspergillus flavus*; antibacterial activity against *Escherichia coli*, *Campylobacter jejuni*, *Salmonella typhimurium*, and *Listeria monocytogenes*; exopolysaccharide production.	PX068363

^a^Sourdough type I refers to naturally fermented mixtures used in artisanal Panettone production, undergoing daily refreshments through back-slopping at 25–35 °C to maintain an active and stable microflora. Dough refers to the leavened mixtures prepared with additional ingredients and collected at specific fermentation stages, including the first doughs after 18 h of fermentation and the final doughs before baking. For gluten-free sourdough, this refers to acidic dough prepared with rice flour

The LAB strains are deposited at the Unimore Microbial Culture Collection (UMCC) and maintained at −80 °C conditions since 2019. These strains were previously studied for their antibacterial and antifungal activities [35] and their ability to produce exopolysaccharides [36–38].

We evaluated both non-lyophilized (NL) and lyophilized cultures of each strain under two pre-freezing conditions (−20 °C and − 80 °C) (Figure S1). Additionally, we assessed the impact on metabolite production and consumption, providing insights on how preservation affects strain performance. SEM analysis was conducted with the aim to evaluate cell surface integrity. This study provides novel perspectives on the customized assessment of freeze-drying protocols for sourdough-derived LAB strains with industrial potential.

## Materials and Methods

### Lactic Acid Bacteria Strains

The five LAB strains tested in this study were *Fructilactobacillus sanfranciscensis* UMCC 2990, *Lactiplantibacillus plantarum* UMCC 2996, *Furfurilactobacillus rossiae* UMCC 3002, *Pediococcus pentosaceus* UMCC 3010 and *Leuconostoc citreum* UMCC 3011 (Table 1). These strains were originally isolated from sourdoughs used in traditional panettone, as described by Iosca et al. 2020. Cultures are deposited at the Unimore Microbial Culture Collection (UMCC; https://www.umcc.unimore.it) of the University of Modena and Reggio Emilia, Italy [39] since 2019 and stored at −80 °C in MRS broth (Oxoid, UK) supplemented with 25% glycerol.

### Freeze-Drying Procedure

An active culture of each strain, regularly propagated every 48 h, was initially grown on MRS plates for two days at 30 °C, under anaerobic conditions. The inoculum consisted of 100µL of the liquid culture; the load was about 10⁷ CFU/ml. Cultures were then harvested from plates using a sterile cotton swab and resuspended in a lyoprotectant solution consisting of 10% skim milk (Oxoid, UK) and MRS broth pH 6.52 (Oxoid, UK), at a 1:1 ratio. Subsequently, 500 µL of each culture suspension was transferred into glass ampoules (Vacule^®^, WHEATON^®^, DWK Life Sciences, Germany).

To maintain sterility and prevent cross-contamination, a cotton plug weighing approximately 20 mg was inserted into each ampoule near the first narrowing. Then, a butane rubber cap (Wheaton^®^ Stopper) was put on the top, avoiding completely closing the ampoules. Two different pre-freezing conditions were followed. The first one consisted of placing the ampoules at 4 °C for 1 h and then at −80 °C for 2 h (protocol PF-80). For the second protocol (PF-20), after taking the ampoules at 4 C° for 1 h, they were placed at −20 °C overnight. Then, they were placed for about 20 h in a Lio 5P freeze dryer (5 Pa, Milan, Italy) equipped with a dry scroll vacuum pump (Edwards, Feldkirchen, Germany). The freeze-drying process was conducted at a default setting of −40 °C and < 0.2 mbar for approximately 20 h. At the end of the freeze-drying, the mechanical stoppering device was screwed to fully close the butane rubbers in a vacuum condition. Finally, each glass ampoule was flame-sealed above the cotton plug using a gas torch to ensure the vacuum inside during long-term storage [38].

### Microbial Counts and Viability Assessment

To check strain viability, colony counts on MRS agar plates were performed by using the strain suspension before freeze-drying (t_0_), and the ampoules immediately after the process (t_1_) or kept at 37 °C for 7 days (t_7_) to approximately simulate 10 years of storage at room temperature, according to previous studies [40–43]. Three ampoules were opened for each strain at the set time point and the dried culture was resuspended in 1 mL of MRS broth. Then, decimal serial dilutions in saline solution (NaCl 0.9%) were made, and 100 µL of appropriate dilutions were plated on MRS agar. Plates were incubated for 48 h at 30 °C, under anaerobic conditions. Microbial counts were converted to log colony-forming units (CFU/mL). To estimate the survival rate (in %), the following equation was used:

Log_10_ CFU/mL, after freeze-drying $\times$ 100

Log_10_ CFU/mL, before freeze-drying

### Fermentative Trial in MRS with the LAB Cultures

All freeze-dried cultures, stored at 37 °C for 7 days (t_7_), and their corresponding non-lyophilized (NL) counterparts were inoculated in triplicate into tubes containing 5 mL of MRS broth. The cultures were initially anaerobically incubated in a 2,5 L anaerobiosis jar (Oxoid) provided with an AnaeroGenTM 2,5 L Atmosphere Generation System (Thermo Scientific) at 30 °C overnight. Subsequently, 2% (v/v) of each pre-culture, adjusted to 0.6 OD_600_ using a spectrophotometer (Jasko V-550, Tokyo, Japan), was transferred into new tubes containing fresh MRS broth. These tubes were then anaerobically incubated at 30 °C, and samples were collected at 5, 15, and 25 h after inoculation. Each sample was analyzed using high-pressure liquid chromatography (HPLC) to determine the concentration of specific metabolites. Additionally, at the conclusion of the fermentation period, the pH of each sample was measured using a pH meter (MicropH 2002, Crison Instrument, Barcelona, Spain).

### Metabolites Detection by HPLC Analysis

The LAB cultures collected during the fermentative trial were centrifuged at 10,000 $\times$ g, 4 °C for ten minutes. The resulting supernatant was diluted and filtered with 0.45 μm PTFE membranes (Sartorius, Göttingen, Germany) in preparation for analysis. 20 µL of each filtered sample were injected into a Jasco LC-Net II/ADC HPLC apparatus (Jasco Inc., Japan) equipped with a Jasco PU-2080 Plus pump. The isocratic elution was carried out using a 300 × 7.8 mm Aminex^®^ HPX-87 H column (Bio-Rad Laboratories, Italy) heated at 40 °C with an Eldex CH-150 oven (Eldex Corp., USA). The mobile phase was composed of H_2_SO_4_ 0.005 N and acetonitrile 5% (v/v) using an operating flow of 0.6 mL/min [44]. Carbohydrates and ethanol were assayed using Jasco UV-2070 Plus (Jasco Inc., Japan); organic acids, 1–3 propandiol, and acetoin concentrations were determined with Jasco RI-2031 Plus detectors (Jasco Inc., Japan). Calibration curves for the standards were generated using Jasco ChromNav software v. 1.18.03 (Tokyo, Japan), which was also used for peak integration and adjustment. The detection limit (LOD) and quantification limit (LOQ) were determined based on the signal-to-noise ratio, where LOD and LOQ correspond to analyte amounts with a signal-to-noise ratio of 3 and 10, respectively [45, 46] (Table S1). All samples were run in triplicate.

### SEM (Scanning Electron Microscopy) Analysis

All NL strains were grown anaerobically at 30 °C overnight in 5 ml of MRS broth. Cells were collected at the logarithmic phase of growth by centrifugation at 10,000 x g for 10 min at 4 °C and washed (three times) with 0.5 mL of sterile 0.1 M phosphate buffer at pH 7.2. Pellets were fixed in 3% glutaraldehyde in phosphate buffer for 2 h, followed by washing several times in 0.1 M phosphate buffer for 15 min intervals. Samples were dehydrated in a series of aqueous ethanol solutions (25%, 50%, 75%, 95% and 100%) for 5 min each [47, 48]. Lyophilized strains were rehydrated by adding 2 mL of sterile deionized water in each tube. The cells were allowed to rehydrate for 10 min at 25 °C with intermittent shaking [49]. Then, cells were treated using the same procedure adopted for NL ones. After fixation and dehydration, samples were mounted on aluminum SEM stubs and sputter-coated with gold. Samples were examined at 10 kV at a pressure of 40 Pa with a Scanning Electron Microscope ESEM Quanta-200 (FEI Company, Oxford Instruments).

### Statistical Analysis

The normality of data was assessed with Shapiro-Wilk Test, performed with R Studio (version 2024.12.0 + 467). Survival rate data after freeze-drying and final pH after 25 h of fermentation were subjected to a one-way analysis of variance (ANOVA) followed by Tuckey’s HSD post hoc test.Data obtained during the fermentative trial were analyzed using the Student’s T-test. Statistical analysis was carried out using IBM SPSS Statistics 20 (IBM, Chicago, IL, USA). Significant differences were considered with p-values ≤ 0.05.

## Results

### Effects of Freeze-Drying on the LAB Strains’ Viability

To evaluate the effectiveness of the preservation methods, the viability of the LAB strains under study was assessed before freeze-drying, immediately after the process (t_1_), and after 7 days of storage at 37 °C (t_7_), simulating long-term storage. Initial bacterial concentrations ranged from approximately 10⁹ CFU/mL to 10⁶ CFU/mL, depending on the strain (Table S2). Table 2 shows the differences in viability after the two pre-freezing protocols. *L. plantarum* UMCC 2996 demonstrated the highest resistance, maintaining its viability after freeze-drying with either protocol, even following simulated aging at 37 °C for 7 days. For *Fu. rossiae* UMCC 3002 and *P. pentosaceus* UMCC 3010, a slight but statistically significant decrease in viability was observed only after the aging period (t_7_). Nonetheless, both strains consistently maintained viability levels above 90%, with the PF-80 protocol being slightly more effective during aging. In contrast, *F. sanfranciscensis* UMCC 2990 and *Le. citreum* UMCC 3011 exhibited more pronounced reductions in viability between t_1_ and t_7_, as well as differences between the two protocols after aging. For *F. sanfranciscensis*, viability remained above 85%, for both PF-20 and PF-80, immediately after freeze-drying. However, after aging, viability significantly declined to 54.45 ± 4.76% with PF-20 and 72.71 ± 1.82% with PF-80. Similarly, *Le. citreum* UMCC 3011 showed reduced viability with the PF-20 protocol at t_1_ (82.15 ± 0.42%), which further declined at t_7_ to 73.89 ± 0.73% (PF-20) and 81.19 ± 1.42% (PF-80). The structural integrity of the freeze-dried samples corroborated these viability results. Ampoules obtained after both pre-freezing protocols (PF-80 and PF-20) exhibited intact, non-collapsed porous and spongy structures resulting from the freeze-drying process (referred to as “cake”) (Figure S2), a morphological feature generally linked to high cell survival [42]. The presence of a cotton plug (with a weight of approximately 20 mg) appeared to contribute to maintaining this structural integrity, as previously suggested by Peiren et al. 2016 [42].

**Table 2:** Viability rate (%) of lyophilized strains subjected to PF-20 and PF-80 pre-freezing protocols, immediately after freeze-drying (t1) and simulated aging (t7)

Strains	PF-20		PF-80	
	t_1_	t_7_	t_1_	t_7_
*F. sanfranciscensis* UMCC 2990	87.28 ± 0.38	55.46 ± 4.76	85.55 ± 0.15	72.72 ± 1.82
*L. plantarum* UMCC 2996	93.93 ± 0.66	94.08 ± 0.94	94.75 ± 0.04	92.01 ± 1.54
*Fu. rossiae* UMCC 3002	93.65 ± 0.24	90.77 ± 2.23	96.69 ± 0.61	94.70 ± 1.15
*P. pentosaceus* UMCC 3010	93.57 ± 0.01	90.62 ± 1.22	94.27 ± 0.09	92.62 ± 0.18
*Le. citreum* UMCC 3011	82.15 ± 0.42	73.89 ± 0.74	89.25 ± 0.43	81.19 ± 1.42

### Effects of Freeze-Drying on Fermentative Aptitude of LAB Strains

#### Evaluation of Sugar consumption, Lactic Acid Production and pH Changes

**Fig. 1:**
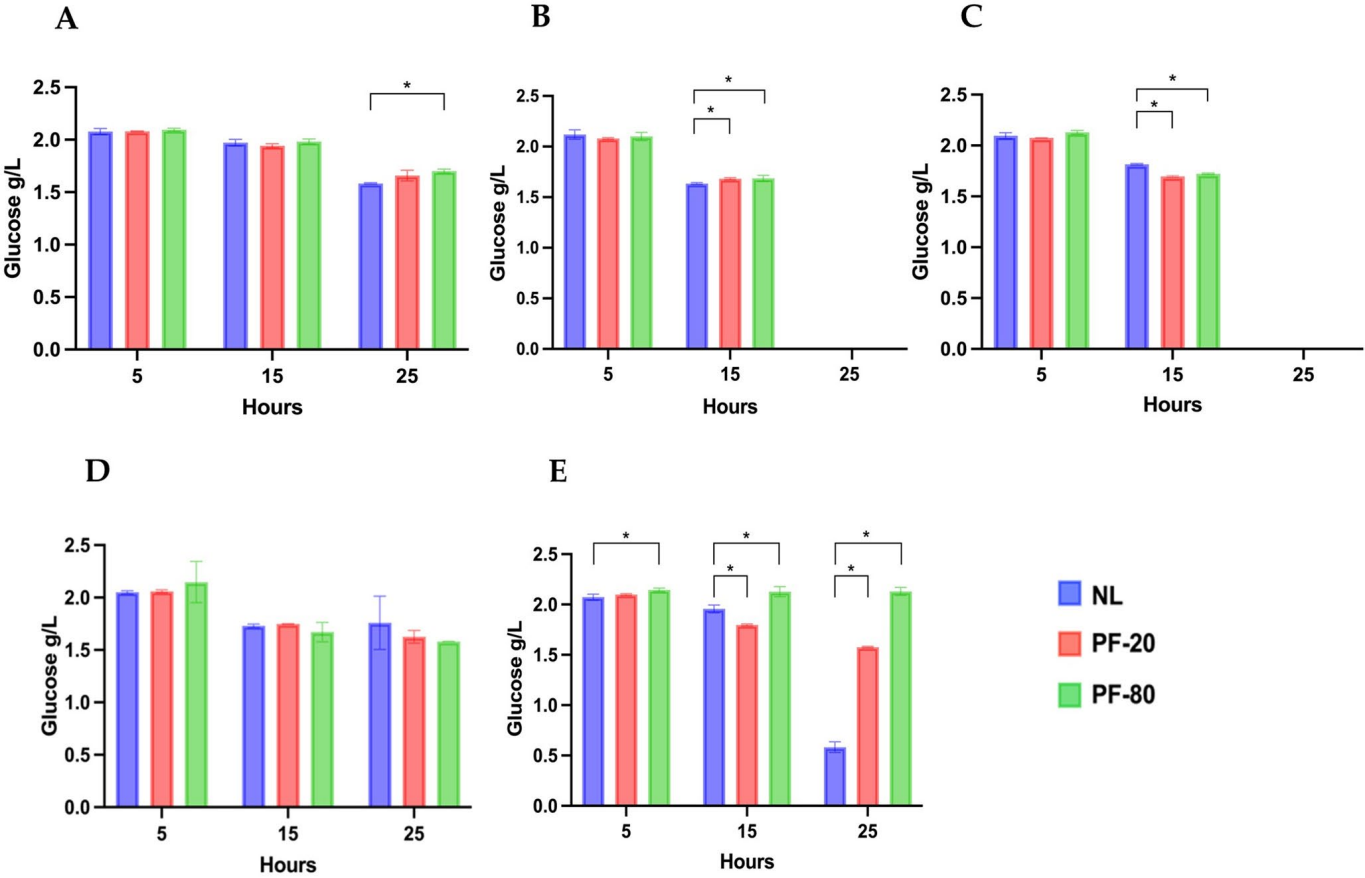
Consumption of glucose (g/L) by non-lyophilized strains (NL) and lyophilized strains with PF-20 and PF-80 pre-freezing protocols. **(a)** *F. sanfranciscensis* UMCC 2990; **(b)** *L. plantarum* UMCC 2996; **(c)** *Fu. rossiae* UMCC 3002; **(d)** *P. pentosaceus* UMCC 3010; **(e)** *Le. citreum* UMCC 3011. Statistical analysis of the data was obtained using Student’s T-test. Significant differences were considered with p-value ≤ 0.05 (*). Error bars are standard deviations (SD) with *n* = 3

**Fig. 2:**
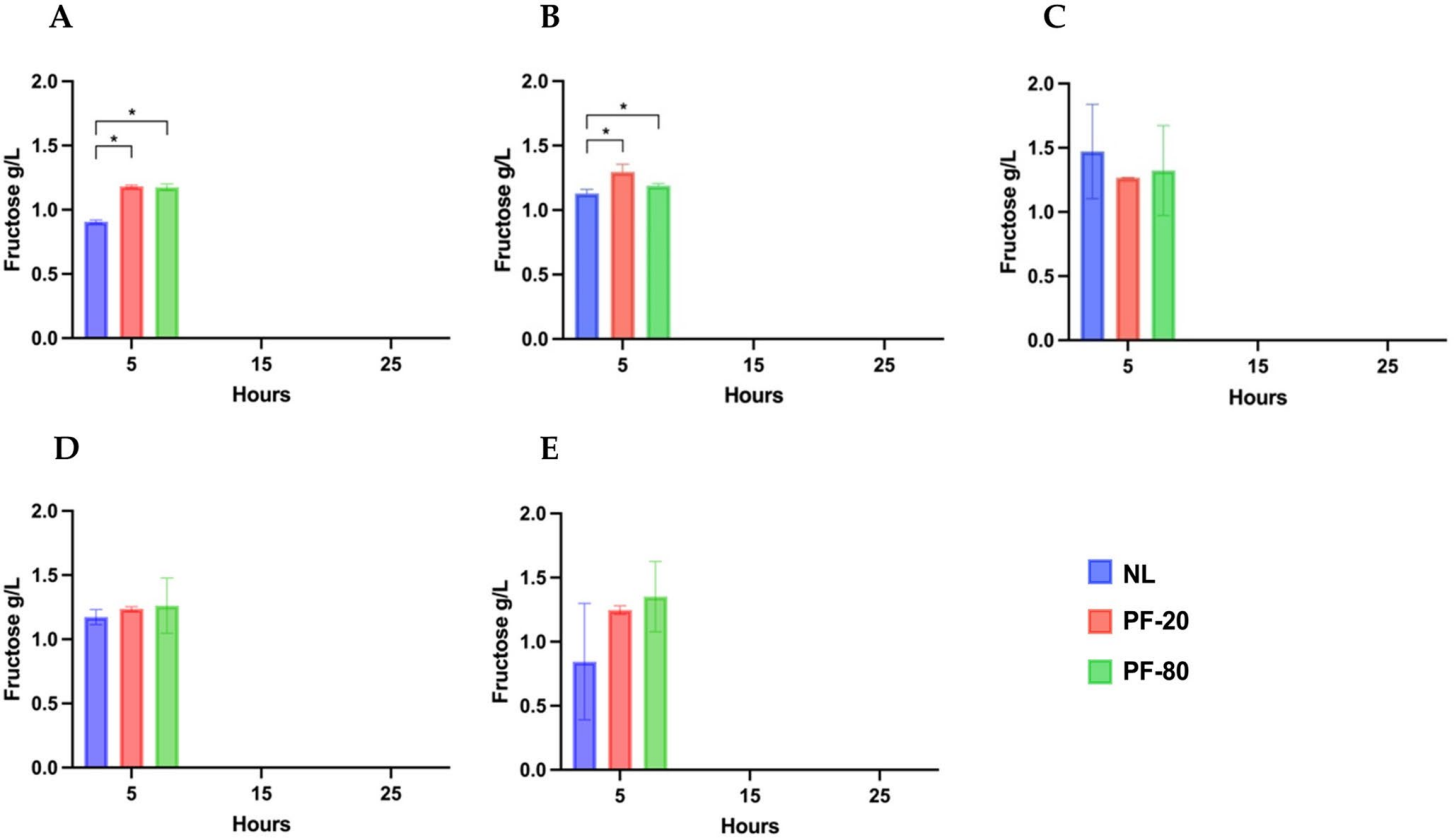
Consumption of fructose (g/L) by non-lyophilized strains (NL) and lyophilized strains with PF-20 and PF-80 pre-freezing protocols. **(a)** *F. sanfranciscensis* UMCC 2990; **(b)** *L. plantarum* UMCC 2996; **(c)** *Fu. rossiae* UMCC 3002; **(d)** *P. pentosaceus* UMCC 3010; **(e)** *Le. citreum* UMCC 3011. Statistical analysis of the data was obtained using Student’s T-test. Significant differences were considered with p-value ≤ 0.05 (*). Error bars are standard deviations (SD) with *n* = 3

**Fig. 3:**
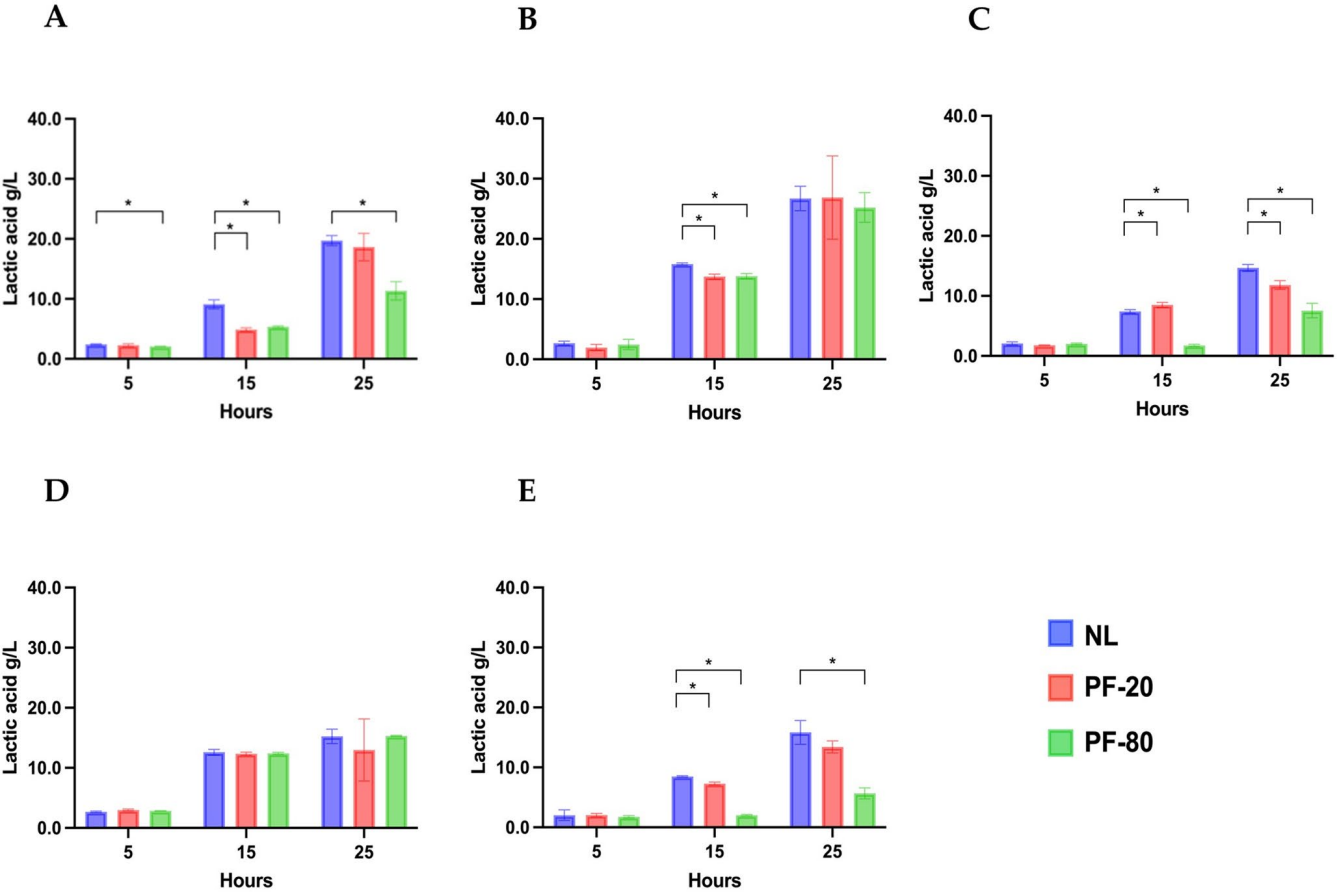
Production of lactic acid (g/L) by non-lyophilized strains (NL) and lyophilized strains with PF-20 and PF-80 pre-freezing protocols. **(a)** *F. sanfranciscensis* UMCC 2990; **(b)** *L. plantarum* UMCC 2996; **(c)** *Fu. rossiae* UMCC 3002; **(d)** *P. pentosaceus* UMCC 3010; **(e)** *Le. citreum* UMCC 3011. Statistical analysis of the data was obtained using Student’s T-test. Significant differences were considered with p-value ≤ 0.05 (*). Error bars are standard deviations (SD) with *n* = 3

Fermentative efficiency was assessed by monitoring glucose (Fig. 1a-e) and fructose consumption (Fig. 2a-e), lactic acid production (Fig. 3a-e) over 25 h of fermentation. Final pH values are reported in Table 3.

**Table 3:** Final pH values measured after 25 h of fermentation for non-lyophilized (NL) and lyophilized LAB strains subjected to two pre-freezing protocols (PF-20 and PF-80). Different letters indicate significant differences between pH values (*p* < 0.05). The values shown are the resulting mean of 3 different replicates ± standard deviation

LAB strain	pH		
	NL	PF-20	PF-80
*F. sanfranciscensis* UMCC 2990	4.52 ± 0.18 ab	4.97 ± 0.23 a	4.60 ± 0.33 b
*L. plantarum* UMCC 2996	4.49 ± 0.27 a	4.73 ± 0.59 a	4.89 ± 0.61 a
*F. rossiae* UMCC 3002	4.75 ± 0.04 a	4.52 ± 0.43 a	4.38 ± 0.29 a
*P. pentosaceus* UMCC 3010	4.09 ± 0.17 a	4.45 ± 0.57 a	4.82 ± 0.48 a
*L. citreum* UMCC 3011	4.80 ± 0.22 a	4.85 ± 0.62 a	5.38 ± 0.35 a

Among the strains tested, *F. sanfranciscensis* UMCC 2990 exhibited a statistically significant difference in residual glucose concentration at 25 h between the non-lyophilized (NL) and lyophilized (PF-80) cultures, with values of 1.58 ± 0.01 g/L and 1.70 ± 0.02 g/L, respectively (Fig. 1a). This suggests a slight reduction in glucose consumption efficiency following lyophilization under the PF-80 condition. For *L. plantarum* UMCC 2996 (Fig. 1b) and *Fu. rossiae* UMCC 3002 (Fig. 1c), glucose consumption trends were comparable between NL and lyophilized cultures, with slight but statistically significant differences at 15 h. Both strains achieved complete glucose consumption by the 25-h mark. Whereas lyophilized cultures of *P. pentosaceus* UMCC 3010 exhibited no significant differences from NL cultures in terms of glucose and fructose consumption or lactic acid production (Fig. 1d).

The most pronounced effect was observed in *Le. citreum* UMCC 3011, where residual glucose levels were significantly higher in both lyophilized conditions (PF-20 and PF-80) compared to the NL culture after 15 h of fermentation (Fig. 1e). At 25 h, residual glucose concentrations were 1.58 ± 0.01 g/L for PF-20 and 2.13 ± 0.03 g/L for PF-80, compared to 0.58 ± 0.04 g/L in the NL culture (Fig. 1e), indicating a marked impairment in fermentative activity post-lyophilization, particularly under the PF-80 condition.

No residual fructose was detected after 15–25 h for any strain, although differences were observed at 5 h (Fig. 2). Significant differences were noted for *F. sanfranciscensis* UMCC 2990 and *L. plantarum* UMCC 2996 (Fig. 2a and b). In *F. sanfranciscensis* UMCC 2990, fructose concentrations in lyophilized cultures were significantly higher (1.18 ± 0.01 g/L for PF-20 and 1.18 ± 0.02 g/L for PF-80) compared to the NL culture (0.91 ± 0.01 g/L). Similarly, *L. plantarum* UMCC 2996 exhibited significantly higher residual fructose levels in lyophilized cultures than in NL cultures, with slightly faster fructose consumption observed under the PF-80 protocol compared to PF-20. For *Fu. rossiae* UMCC 3002, *P. pentosaceus* UMCC 3010, and *Le. citreum* UMCC 3011, fructose consumption showed no statistically significant differences at this time, despite some variability (Fig. 2c, d and e).

Regarding lactic acid production (Fig. 3) by the tested strains, lyophilized cultures of the heterofermentative strains *F. sanfranciscensis* UMCC 2990 exhibited reduced lactic acid production compared to NL cultures at both 15 and 25 h of fermentation. The PF-80 protocol further exacerbated the decline in lactic acid synthesis (Fig. 3a). For *L. plantarum* UMCC 2996, a facultative heterofermentative LAB, while the NL culture exhibited slightly higher lactic acid levels at 15 h, no significant differences were observed by the end of 25 h (Fig. 3b). Notably, this strain displayed the highest lactic acid production across all conditions. *Fu. rossiae* UMCC 3002, an obligate heterofermentative LAB, displayed markedly lower lactic acid levels under the PF-80 protocol compared to NL cultures at both 15 and 25 h (Fig. 3c). No statistically significant differences were observed for *P. pentosaceus* UMCC 3010, a homofermentative strain (Fig. 3d). Notably, *Le. citreum* UMCC 3011 under the PF-80 protocol exhibited reduced lactic acid production, as evidenced by a higher pH value after 25 h compared to other conditions and strains. All other strains demonstrated a marked pH reduction from the initial value of 6.52 pH (Table 3).

#### Evaluation of Organic Compounds

**Fig. 4:**
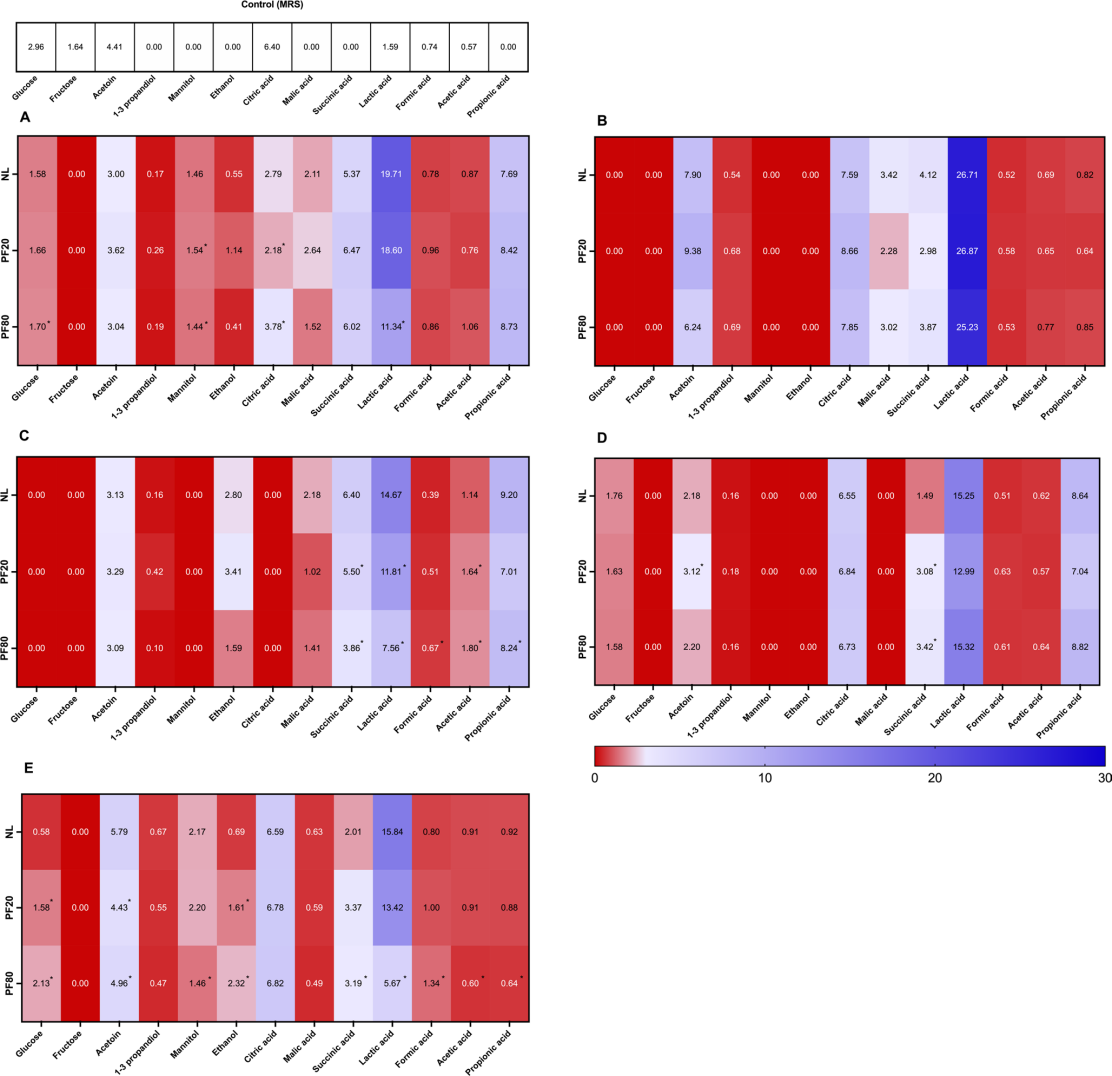
Different compounds (g/L) detected by HPLC and pH values after 25 h of the fermentation trial in MRS broth by using the lyophilized and non-lyophilized LAB cultures, including unfermented MRS.^1^ **(A)** *F. sanfranciscensis* UMCC 2990; **(B)** *L. plantarum* UMCC 2996; **(C)** *Fu. rossiae* UMCC 3002; **(D)** *P. pentosaceus* UMCC 3010; **(E)** *Le. citreum* UMCC 3011 ^1^ The measurement was carried out by three independent experiments, and the Student’s T-test was performed to detect the significant variations comparing the data obtained from lyophilized strains with PF-20 and PF-80 protocols to non- lyophilized strains (NL). The significance for *p* < 0.05 is marked with an asterisk (*)

In this study, the impact of lyophilization and pre-freezing conditions on the metabolic activity of the tested LAB strains was assessed by profiling organic acids and related metabolites. *F. sanfranciscensis* UMCC 2990 exhibited significant changes in mannitol, citric acid, and lactic acid content after lyophilization (Fig. 4a), likely reflecting disruptions in central carbon metabolism—particularly in pyruvate conversion and redox processes sensitive to membrane and enzyme stability. On the contrary, for *L. plantarum* UMCC 2996, no significant differences in organic acid profiles were observed between non-lyophilized (NL) and lyophilized cultures (Fig. 4b), indicating a stable metabolic output and high resilience to freeze-drying. This suggests its potential suitability for industrial applications. *Fu. rossiae* UMCC 3002 displayed broad variability in its organic acid profile (Fig. 4c). Under PF-80 pre-freezing, all acids except citric and malic were significantly affected, whereas PF-20 influenced only succinic, acetic, and lactic acids. *P. pentosaceus* UMCC 3010 showed a marked increase in succinic acid in lyophilized samples (Fig. 4d), suggesting an altered metabolic response to freezing-induced stress. *Le. citreum* UMCC 3011 demonstrated the most extensive metabolic variation. The PF-80 lyophilized culture showed significant alterations in acetoin, mannitol, ethanol, and multiple organic acids (succinic, lactic, formic, acetic, and propionic) (Fig. 4e), highlighting its sensitivity to preservation stress and the potential impact on its fermentative and sensory properties. Overall, the findings reveal specific responses to lyophilization and underscore the importance of customized preservation strategies to maintain the functional attributes of LAB strains for both culture collections and industrial use.

### Effects of freeze-drying on the LAB Cell Surface by SEM Analysis

**Fig. 5:**
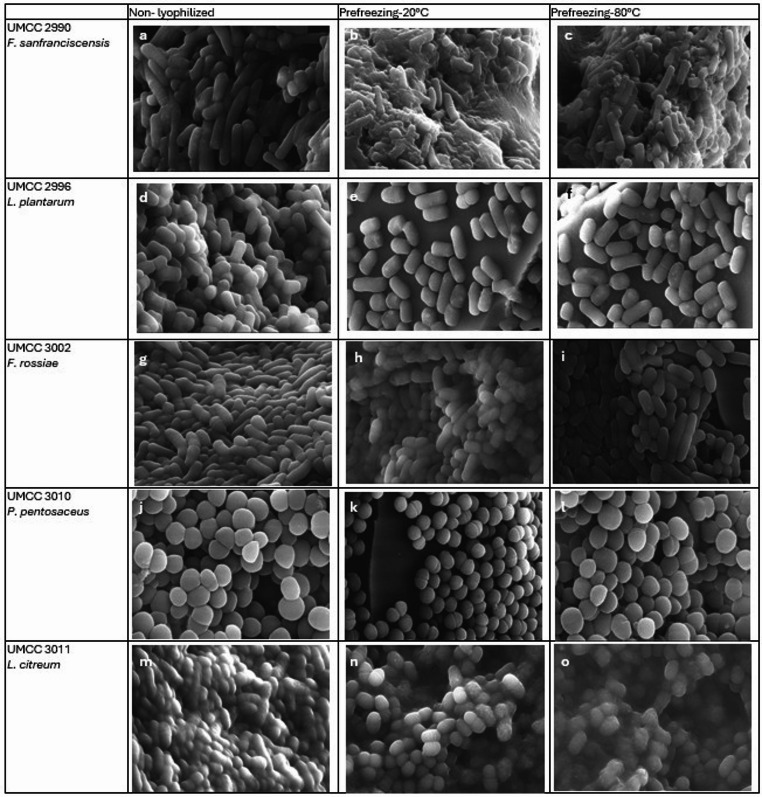
Images acquired from SEM; *F. sanfranciscensis* UMCC 299 non-lyophilized strains (NL) (**a**), lyophilized strains with PF-20 (**b**) and PF-80 pre-freezing protocols (**c**). *L. plantarum* UMCC 2996 non-lyophilized strains (NL) (**d**), lyophilized strains with PF-20 (**e**) and PF-80 pre-freezing protocols (**f**). *Fu. rossiae* UMCC 3002 non-lyophilized strains (NL) (**g**), lyophilized strains with PF-20 (**h**) and PF-80 pre-freezing protocols (**i**). *P. pentosaceus* UMCC 3010 non-lyophilized strains (NL) (**j**), lyophilized strains with PF-20 (**k**) and PF-80 pre-freezing protocols (**l**). *Le. citreum* UMCC 3011 non-lyophilized strains (NL) (m), lyophilized strains with PF-20 (**n**) and PF-80 pre-freezing protocols (**o**)

SEM images revealed notable macroscopic differences in cellular morphology among the tested strains depending on the pre-freezing protocol and simulated aging (Figs. 5a-o).

Pronounced cell alterations were observed in *F. sanfranciscensis* UMCC 2990 (Figs. 5a, b and c) and *Le. citreum* UMCC 3011 (Figs. 5m, n, o). These strains exhibited visibly wrinkled cells surrounded by cellular debris, indicating partial structural alteration. The debris were particularly abundant in *F. sanfranciscensis* UMCC 2990, under both pre-freezing conditions, suggesting a higher sensitivity to freeze-drying. Interestingly, the pre-freezing at −80 °C appeared to be more critical for *Le. citreum* UMCC 3011 (Fig. 5o), while *F. sanfranciscensis* UMCC 2990 showed greater damage under the − 20 °C condition (Fig. 5b). These morphological observations are consistent with the viability data, reinforcing the strain-specific impact of pre-freezing temperature on cellular integrity during lyophilization.

*L. plantarum* UMCC 2996 exhibited no discernible morphological differences between the NL and lyophilized samples, regardless of the pre-freezing temperature, indicating a high structural resilience to the freeze-drying process (Figs. 5d, f).

In contrast, *P. pentosaceus* UMCC 3010 and *F. rossiae* UMCC 3002 displayed slightly more wrinkled cell surfaces after lyophilization with a pre-freezing step at −20 °C, although no signs of cell lysis were observed (Figs. 5g, h, i). This suggests moderate stress without compromising membrane integrity.

## Discussion

Microorganisms represent a significant portion of biodiversity across all ecological habitats, profoundly impacting ecosystem functions, human health, and various industrial and biotechnological applications, including the food industry. In this context, microorganisms play an essential role in enhancing product quality, optimizing processing efficiency, extending shelf life, and contributing to waste reduction. These diverse applications emphasize the necessity of efficiently preserve the exploitable microbial diversity. Among the various preservation techniques, cryopreservation and freeze-drying are widely acknowledged as the most effective strategies for maintaining the long-term stability of microorganisms [24]. In alignment with the guidelines set forth by the World Federation for Culture Collections (WFCC), both methods are recommended for each culture to guarantee reliable preservation [23, 24]. Microbial strains exhibit varying sensitivities due to intrinsic characteristics, highlighting the need for strain-specific protocols that account for suspension media, protectants, cell concentrations, and optimal cooling rates [28, 53]. Validation of these protocols is equally essential to guarantee the reproducibility, purity, identity, and stability of preserved biological materials over time [34]. Freeze-drying, despite being highly effective, remains a complex and strain-dependent process. Its success depends on factors such as freezing temperature, initial cell concentration, and the choice of protectants, as these parameters significantly impact cell viability by influencing membrane integrity and permeability [29, 50–53]. Optimized procedures are thus essential to preserve the viability and phenotypic traits of microorganisms after rehydration.

In this study, we investigated five LAB strains of UMCC culture collection. These strains are known for their antibacterial, antifungal activities, and exopolysaccharide production [37–39], making them promising for starter cultures design. We evaluated how these strains respond to two different freeze-drying protocols. Viability and fermentative performance were assessed for both NL and lyophilized strains using two pre-freezing temperatures. Measurements were taken immediately after freeze-drying and after seven days of storage at 37 °C to approximately simulate prolonged aging. Additionally, we simulated fermentation durations to approximate non-lyophilized conditions and compared the two freeze-drying methods in terms of metabolite production and consumption. Cell viability after freeze-drying varied among strains, with cells harvested during the stationary phase displaying greater resistance to environmental stresses compared to those harvested in lag or exponential phases [54]. However, even under similar initial conditions, *F. sanfranciscensis* UMCC 2990 and *Le. citreum* UMCC 3011 exhibited extended lag phases and lower growth, which likely contributed to their reduced survival rates. Their sensitivity to freeze-drying seems to be confirmed by SEM membrane integrity observations. UMCC 2990 proved to be the most sensitive strain, particularly after simulated aging, whereas *L. plantarum* UMCC 2996 demonstrated the highest resilience and stability across all tested conditions, showing also comparable morphologies for all samples under SEM observation. In terms of fermentative performance, we focused on glucose and fructose consumption, lactic acid production, and pH reduction which are key parameters in bio preservation of fermented foods [55]. Slight variability in metabolite content was observed between NL and lyophilized cultures, except for *L. plantarum* UMCC 2996, which showed no significant changes and achieved the highest lactic acid production across all conditions. Conversely, significant differences were observed for *F. sanfranciscensis* UMCC 2990 and *Le. citreum* UMCC 3011, likely due to their lower viability post-freeze drying. For *F. sanfranciscensis*, the ability to use fructose as an external electron acceptor, producing mannitol [56, 57], may explain its incomplete glucose consumption in MRS medium and the significant differences in mannitol production between NL and lyophilized cultures. A similar trend was observed in *Le. citreum* UMCC 3011, where mannitol production was significantly lower in the PF-80 lyophilized cultures. The PF-80 protocol showed reduced fermentative stability, as indicated by higher residual glucose and pH levels, lower lactic acid production, and a shift towards ethanol synthesis. These results align with the known sensitivity of *Leuconostoc* species to low temperatures, particularly without gradual adaptation cycles during cultivation [58]. Rapid temperature drops may disrupt exopolysaccharide synthesis, leading to altered carbohydrate metabolism and reduced glucose consumption, particularly in PF-80 lyophilized cultures. Overall, these findings underscore the importance of optimizing preservation protocols to maintain the viability and functionality of microbial strains. Tailored approaches that consider both intrinsic strain characteristics and extrinsic process parameters are essential for effective long-term preservation in culture collections (CCs) and microbial biorepositories (mBRCs). The observed differences in viability and fermentative performance among the LAB strains following freeze-drying likely could be related to a combination of intrinsic physiological traits and strain-specific responses to preservation stress. Factors such as cell wall composition, membrane lipid profiles, exopolysaccharide production, and growth phase can significantly influence a strain’s ability to withstand dehydration and rehydration. For instance, strains like *L. plantarum* UMCC 2996, which demonstrated high resilience, may possess more robust membrane structures or higher levels of protective solutes that stabilize cellular components during freeze-drying. Conversely, the sensitivity of *F. sanfranciscensis* UMCC 2990 and *Le. citreum* UMCC 3011 may be attributed to less efficient stress response systems or membrane compositions more prone to damage from ice crystal formation and osmotic stress.

## Conclusions

This study evaluated the long-term preservation performance of five LAB strains from the UMCC culture collection, focusing on their viability and retention of industrially relevant phenotypic traits following freeze-drying, under two pre-freezing protocols (PF-20 and PF-80). Among the tested strains, *L. plantarum* UMCC 2996 exhibited the highest resilience, maintaining both viability and fermentative capacity across all conditions. In contrast, *F. sanfranciscensis* UMCC 2990 and *Le. citreum* UMCC 3011 showed greater sensitivity to freeze-drying, particularly after simulated aging, with notable decline in viability and fermentative performance. It is noteworthy that the study highlights that, while PF-80 generally offered better viability preservation, it was not always optimal. For certain strains, such as *Le. citreum*, PF-80 was associated with reduced metabolic activity and altered fermentation profiles, suggesting that rapid freezing may disrupt cellular integrity in sensitive strains.

Although this study is limited to a few LAB strains from 5 different species, results support the hypothesis that freeze-drying can impart different effects on morphological and physiological traits of strains. Further studies can provide insights into the complex dynamics of long-term storage effects under freeze-drying. In particular, we believe that enlarging the number of considered strains and integrating metatranscriptomic analysis would provide deeper insights into cellular stress responses and molecular mechanisms. SEM analysis can be also supported by flow cytometry, for a quantitative cellular damage evaluation.

## Supplementary Information

Below is the link to the electronic supplementary material.

Supplementary Material 1

## Data Availability

All data generated or analyzed during this study are included in this published article and its supplementary information files. Additional datasets are available from the corresponding author upon reasonable request.

## Code Availability

Not applicable. No custom code or software was developed or used in this study.
