# Supplementary material for: Freeze-Drying Effects on Viability and Cellular Stability in a Subset of Sourdough Lactic Acid Bacteria Strains
Source: Curr Microbiol. 2025 Dec 11;83(1):76. doi: 10.1007/s00284-025-04673-5 (PMC12698733; doi:10.1007/s00284-025-04673-5)
Supplement: Supplementary file 1 — Supplementary Material 1 [file 284_2025_4673_MOESM1_ESM.docx]

**File 7. Supplementary Data**

**Freeze-drying effects on viability and cellular stability in a subset of sourdough lactic acid bacteria strains**

**Current Microbiology (CMIC)**

**Valentina Musi^1^, Elisa Aiello^1^, Mattia Pia Arena^1^, Luciana De Vero^1^, Andrea Pulvirenti^1^ Maria Gullo^1^***

Department of Life Sciences, University of Modena and Reggio Emilia, 42122 Reggio Emilia, Italy

*****Correspondence: [maria.gullo@unimore.it](mailto:maria.gullo@unimore.it)


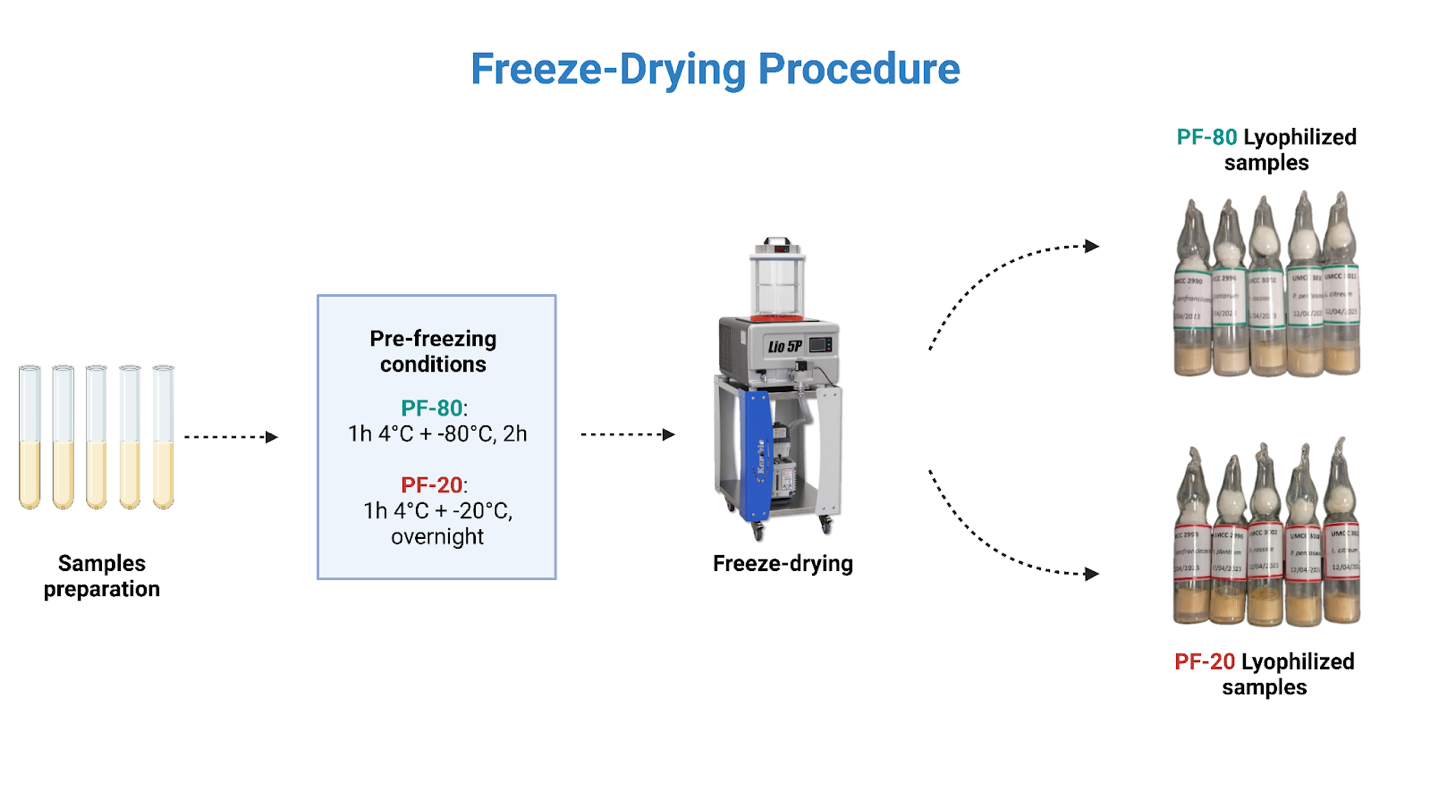


**Figure S1.** Freeze-drying procedure for the preservation of LAB strains using two different pre-freezing conditions: PF-80 and PF-20. Figure created in [BioRender.com](http://biorender.com/).


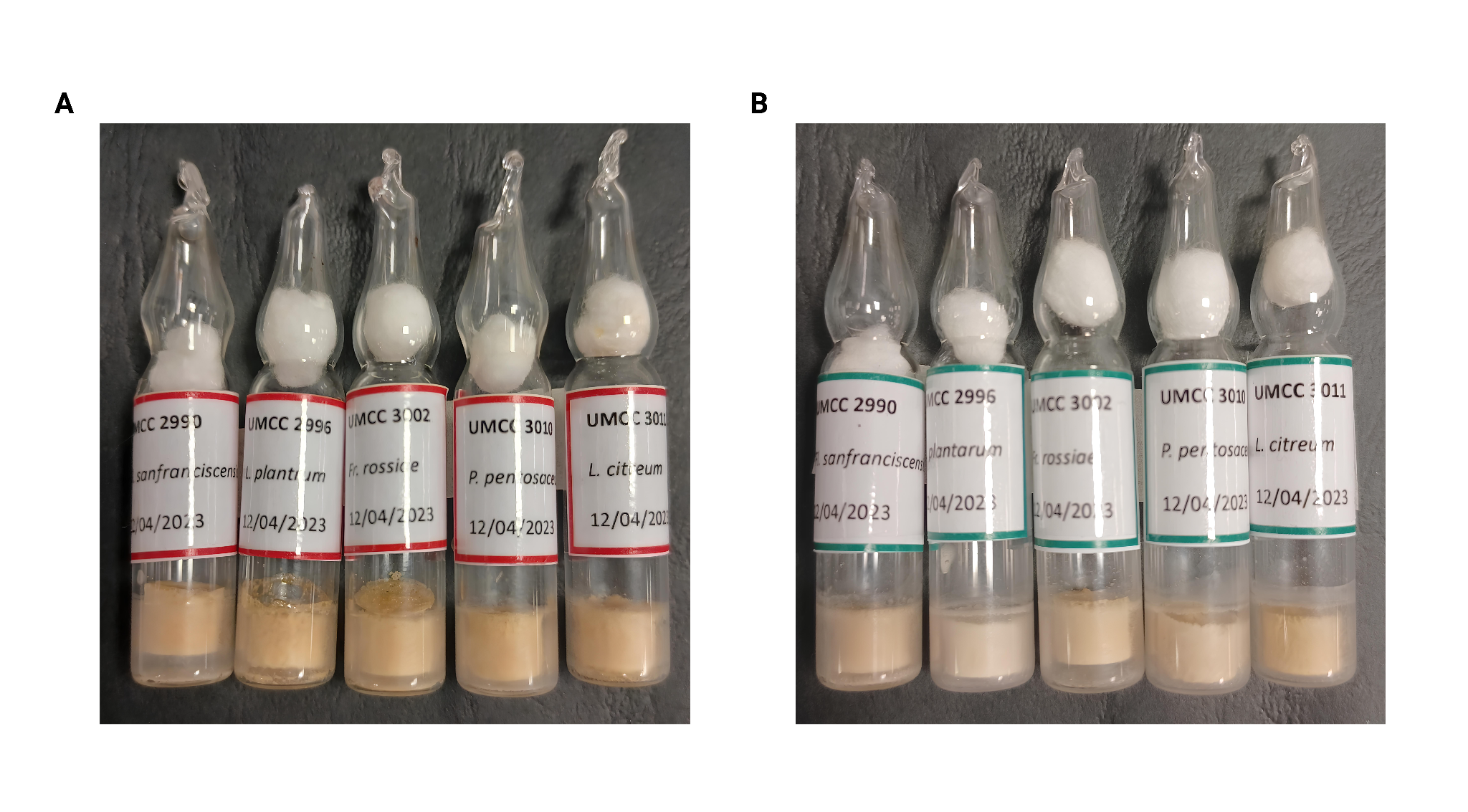


**Figure S2.** Lyophilized ampoules of the selected LAB obtained with PF-20 protocol **(A)** and PF-80 protocol **(B).**

| **Analyte** | **LOD g/L** | **LOQ (g/L)** |
| --- | --- | --- |
| Glucose | 0,4477098 | 1,492366 |
| Fructose | 0,6888212 | 2,296071 |
| Acetoin | 0,09193119 | 0,3064373 |
| 1-3 propandiol | 0,04116704 | 0,1372235 |
| Mannitol | 0,1622332 | 0,5407773 |
| Ethanol | 4,364248 | 14,54749 |
| Citric acid | 0,003706786 | 0,01235595 |
| Malic acid | 0,01834251 | 0,06114171 |
| Succinic acid | 0,01519075 | 0,05063585 |
| Lactic acid | 0,01228293 | 0,04094309 |
| Formic acid | 0,02863853 | 0,09546178 |
| Acetic acid | 0,02768051 | 0,09226837 |
| Propionic acid | 0,002083003 | 0,006943344 |

**Table S1.** LOD and LOQ of the HPLC detected compounds

| **Strains** | **PF-20** | | | **PF-80** | | |
| --- | --- | --- | --- | --- | --- | --- |
|  | ***t_0_*** | ***t_1_*** | ***t_7_*** | ***t_0_*** | ***t_1_*** | ***t_7_*** |
| ***F. sanfranciscensis* UMCC 2990** | 8.52±0.03 | 7.44±0.06 | 4.73±0.42 | 8.22±0.03 | 7.04±0.04 | 5.98±0.17 |
| ***L. plantarum* UMCC 2996** | 9.48±0.07 | 8.91±0.01 | 8.92±0.16 | 9.69±0,01 | 9.19±0,01 | 8.92±0.16 |
| ***F. rossiae* UMCC 3002** | 9.16±0.03 | 8.58±0.05 | 8.31±0.23 | 9.37±0.01 | 9.06±0.06 | 8.87±0.15 |
| ***P. pentosaceus* UMCC 3010** | 9.47±0.01 | 8.86±0.01 | 8.58±0.13 | 9.73±0.02 | 9.17±0.03 | 9.01±0.04 |
| ***L. citreum* UMCC 3011** | 7.59±0.05 | 6.24±0.01 | 5.61±0.09 | 9.05±0.04 | 8.08±0.08 | 7.35±0.16 |

**Table S2.** Colony counts on the MRS agar. Data are expressed as log_10_ of colony forming unit CFU/mL before freeze-drying process (t_o_), after freeze-drying process (t_1_); after 7 days at 37°C simulating 10 years aging (t_7_). Values are mean ± standard deviation of three replicates.
